# The weight-adjusted-waist index predicts all-cause and cardiovascular mortality in general US adults

**DOI:** 10.1016/j.clinsp.2023.100248

**Published:** 2023-07-11

**Authors:** Ying Han, Jieli Shi, Pengfei Gao, Lin Zhang, Xuejiao Niu, Na Fu

**Affiliations:** Department of Endocrinology and Metabolism (Rheumatism and Immunity), Haibin People's Hospital, Tianjin Binhai New Area, PR China

**Keywords:** Weight-adjusted-waist index, All-cause death, Cardiovascular death, Mortality, NHANES

## Abstract

**Background:**

The authors examined the relationship between Weight-adjusted Waist Index (WWI) and all-cause and cardiovascular mortality among adults in the US.

**Methods:**

This prospective cohort study included 26,882 individuals who participated in the National Health and Nutrition Examination Survey (NHANES) from 2005 through 2014. WWI was calculated as waist circumference divided by the square root of weight. The main outcomes of this study were all-cause mortality and cardiovascular mortality. Mortality status and cause of death were determined by NHANES-linked National Death Index records through December 31, 2015. Cox proportional hazard models and Kaplan-Meier analysis were used to estimate Hazard Ratios (HR) and 95% CIs for mortality for all causes and cardiovascular diseases.

**Results:**

A total of 26,882 participants with a mean WWI of 10.89 ± 0.01, of whom 49.23% were male. The average follow-up time was 68.95 ± 1.07 months, and 1870 participants were determined as deceased (4.99%), including 349 cardiovascular death (0.88%). The Kaplan-Meier analysis demonstrated a significant difference in all-cause and cardiovascular mortality between patients with WWI <11.33 and ≥11.33 (both log-rank test *p* < 0.0001). The fully adjusted Cox proportional hazard model indicated that a higher WWI level (≥ 11.33) was associated with an increased 95% risk for cardiovascular mortality (HR = 1.95, 95% CI 1.30‒2.93) and 68% risk for all-cause death (HR = 1.68, 95% CI 1.41‒2.00) compared with the counterparts.

**Conclusions:**

Elevated WWI levels were associated with a higher risk of cardiovascular mortality and all-cause mortality independently.

## Highlights

• WWI was a potential predictor for cardiovascular mortality with a cut-off of 11.33.
• Higher WWI associated with an increased 95% risk for cardiovascular mortality.
• Higher WWI associated with an increased 68% risk for all-cause mortality.

## Introduction

The condition of obesity is characterized by an excessive buildup of adipose tissue that has deleterious implications for individual health. Over the period spanning 1980 to 2013, the global incidence of obesity has risen by 27.5% among adults and 47.1% among children.^1^ Obesity has become a worldwide epidemic and causes huge health burdens for both individuals and society. It is linked to a range of health conditions, including cardiovascular disease (CVD), hypertension, type 2 diabetes mellitus (T2DM), hyperlipidemia, stroke, osteoarthritis and so on.^2^ Compared with normal-weight individuals, the obese population has a higher all-cause mortality.^3^ Furthermore, it engenders an economic encumbrance on society, as evidenced by a surge in yearly healthcare expenses by 36% and medication expenditures by 77% when compared to individuals of average weight. The medical expenditure panel surveys (MEPS) yielded findings indicating that patients classified as obese incurred expenses that were $600 greater annually when compared to patients of normal weight.^4^

In 2018, Park et al. introduced the weight-adjusted-waist index (WWI) as a novel anthropometric index for obesity. The index was derived by dividing waist circumference (WC) by the square root of weight, which demonstrated the ability to predict the risk of cardiometabolic diseases and mortality in the Korean population.^5^ Research has indicated that WWI serves as a more reliable prognosticator of hypertension incidence compared to measures of body mass index (BMI) and WC.^6^ The occurrence of diabetes was also found to be significantly higher in individuals who had higher WWI, indicating an independent association between the two variables.^7^ Previous research has demonstrated that WWI serves as an anthropometric measure that exhibits a positive correlation with fat mass and a negative correlation with muscle mass among older adults.^8^ These findings imply that WWI may serve as a reliable and comprehensive indicator of obesity.

The World Health Organization's BMI has been a longstanding measure for evaluating obesity. WWI is a relatively new index that has not yet been extensively studied in relation to cardiovascular and all-cause mortality in the US population. Therefore, the objective of this study was to investigate the association between WWI and mortality in the general adult population of the United States, utilizing data from national health and nutrition examination survey (NHANES).

## Methods

### *Survey population*

NHANES is a recurring, nationally representative study that employs a complex multistage probability design to assess the nutritional and health status of the non-institutionalized civilian population in the United States.^9^ Participants are interviewed in their homes and provide standardized responses regarding their demographic, socioeconomic, health-related behaviors, and health information. Trained medical professionals conduct physical and laboratory examinations in a Mobile Examination Center (MEC).

The NHANES study protocols were approved by the Research Ethics Review Board of the National Center of Health Statistics, and written informed consent was obtained from all survey participants (Protocol number of NHANES 2005‒2010: Protocol #2005–06; Protocol number of NHANES 2011‒2014: Protocol #2011–17). The comprehensive NHANES study designs and data are accessible to the public at www.cdc.gov/nchs/nhanes/. This study adhered to the Strengthening the Reporting of Observational Studies in Epidemiology (STROBE) reporting guidelines for cross-sectional studies.^10^

**Fig. 1:**
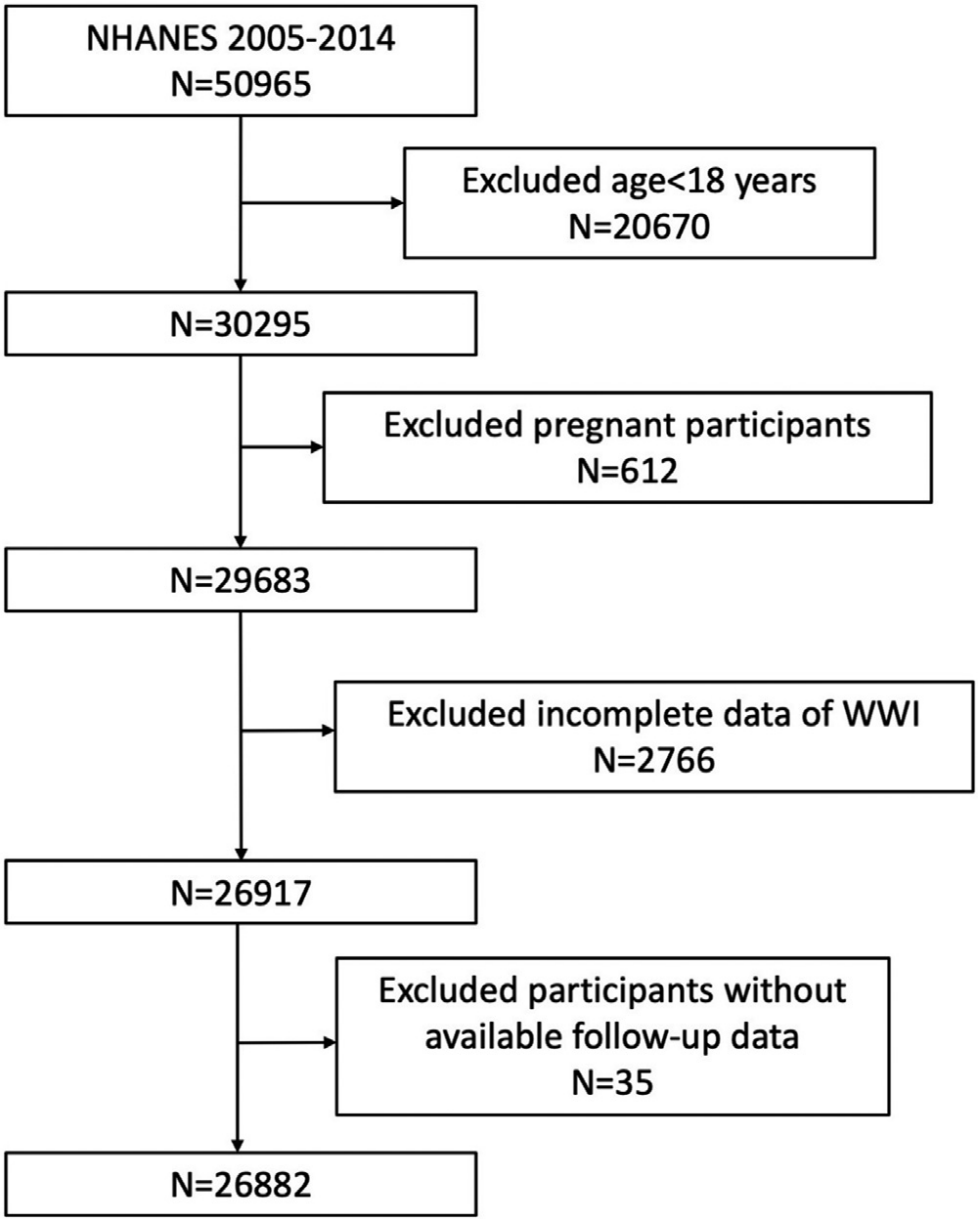
Flowchart of the sample selection from NHANES 2005‒2014.

This study utilized data from the NHANES survey cycle spanning from 2005 to 2014, which encompassed a total of 50,965 individuals from the United States. Participants who were at least 18 years of age and had complete data regarding WWI and mortality status were included in the analysis. After the exclusion of participants aged < 18 years (*n* = 20,670), pregnant (*n* = 612), missing the data about WWI (*n* = 2766) and mortality status (*n* = 35), 26,882 eligible participants were included in the final analysis, which represented 212.45 million noninstitutionalized residents of the United States (Fig. 1).

### *Assessment of weight-adjusted-waist index*

In the present study, the anthropometric index known as WWI was utilized to estimate obesity based on measurements of WC and weight. A higher WWI score was indicative of a greater degree of obesity. Trained health technicians collected data on WC and weight measurements from study participants in the MEC. The WWI for each participant was calculated by dividing their WC measurement in centimeters by the square root of their weight measurement in kilograms and then rounding the result to two decimal places. WWI was employed as an exposure variable in the present study.

### *Other variables definitions*

The demographic covariates examined in this study encompassed gender (male/female), age (40‒49/ 50‒59/ 60‒69/ > 69 years), race (Mexican American/other Hispanic/non-Hispanic White/non-Hispanic Black/other races), an education level (less than high school/ high school or general educational development/above high school/others) and smoking status (never/ever/current/unknown). Several anthropometric and laboratory covariates also have been incorporated, including body mass index (BMI, kg/m^2^, calculated as weight in kilograms divided by height in meters squared), systolic blood pressure (SBP, mmHg), diastolic blood pressure (DBP, mmHg), urinary albumin (mg/L), urinary creatinine (mg/dL), alanine transaminase (ALT, U/L), aspartate transaminase (AST, U/L), serum creatinine (mg/dL), serum uric acid (μmol/L), total cholesterol (mmol/L), triglycerides (mmol/L), fasting plasma glucose (uIU/mL), high-density lipoprotein-cholesterol (HDL-C, mmol/L) and low-density lipoprotein-cholesterol (LDL-C, mmol/L). The study incorporated the health status variables of hypertension (yes/no), and diabetes (yes/no) were also included. Diabetes was operationally defined as either self-reported, physician-diagnosed diabetes or a hemoglobin A1c level ≥ 6.5%. Hypertension was operationally defined as either self-reported, physician-diagnosed hypertension or three consecutive systolic blood pressure measurements ≥ 140 mmHg or diastolic blood pressure ≥ 90 mmHg. The comprehensive measurement processes for these variables are readily accessible to the public via the website www.cdc.gov/nchs/nhanes/.

### *All-cause and cardiovascular disease mortality*

The NHANES dataset is associated with the National Death Index's death certificate records. This dataset includes mortality information, such as follow-up duration and underlying cause of death, for individuals aged 18 years or older until December 31st, 2015. Cardiovascular Death (CVD) was defined as any death resulting from heart disease or cerebrovascular disease, as indicated by the International Statistical Classification of Diseases and Related Problems, Tenth Revision codes I00 to I09, I11, I13, I20 to I51, and I60 to I69. The linkage method and analytical guidelines are thoroughly described and publicly accessible at www.cdc.gov/nc.

### *Statistical analysis*

Due to the intricate sampling design of NHANES, all statistical analyses were conducted by incorporating sample weights, clustering, and stratification in accordance with CDC analytic guidelines. Descriptive analyses utilized either a weighted Student's *t*-test (for continuous variables) or a weighted Chi-Square test (for categorical variables) to assess differences among participants categorized by mortality status. The means with standard error (SE) are used to summarize continuous variables, while proportions are utilized to present categorical parameters. To determine the optimal cut-off, point of WWI in discriminating the occurrence of cardiovascular death, a receiver operating characteristic (ROC) curve with an Area Under the Curve (AUC) was generated. Subsequently, the study cohort was stratified based on the WWI value (WWI cut-off = 11.33). The Cox proportional hazards model was employed to compute hazard ratios (HRs) and 95% Confidence Intervals (95%CIs) to evaluate the correlation between WWI with all-cause mortality and cardiovascular mortality. The reference group was defined as the cohort with WWI < 11.33. Three regression models were developed to facilitate statistical inference. Model 1 only comprised WWI (categorical), Model 2 encompassed demographic variables such as age, gender, and race, and Model 3 comprised variables from Model 2 in addition to education level, urinary albumin, urinary creatinine, ALT, AST, total cholesterol, serum creatinine, triglycerides, serum uric acid, BMI, SBP, DBP, fasting plasma glucose, HDL-C, LDL-C, smoking, hypertension, and diabetes status. Kaplan-Meier curves and log-rank tests were further generated to assess all-cause mortality and cardiovascular mortality across WWI categories with the adjustment of all variables. Multiple imputations were employed to address missing values in the variables. The statistical analyses were conducted using R version 4.1.3 (www.R-project.org, The R Foundation) and Empower software (www. empowerstats.com; X&Y Solutions, Inc., Boston MA). A two-sided *p* < 0.05 was considered statistically significant.

## Results

### *Baseline characteristics of participants*

A total of 26,882 participants with an average (±SE) follow-up length of 68.95 ± 1.07 months were enrolled, of whom 49.23% were male and 50.77% were female. The mean WWI was 10.89 ± 0.01 overall. Participants who were still alive were younger, were more frequently female, had higher lower educational levels and were more likely to a non-smokers than those who were deceased. Participants who alive also had a lower prevalence of diabetes and hypertension, a higher DBP, urinary creatinine, total cholesterol, LDL-C level, a lower SBP, urinary albumin, AST, serum creatinine, serum uric acid and fasting plasma glucose levels. The average of WWI was higher among adults who were deceased compared to those who were alive (11.46 ± 0.03 vs. 10.86 ± 0.01, *p* < 0.0001) (Table 1).

**Table 1:** Baseline characteristics of participants who were still alive versus those who were deceased by December 31th, 2015.

	Overall	Still alive, *N* = 25,012	Deceased, *N* = 1870	P value
Age (years),% (SE)				
18–39	38.80 (0.70)	40.42 (0.72)	7.98 (0.88)	<0.0001
40–59	37.73 (0.48)	38.53 (0.50)	22.47 (1.38)	
≥ 60	23.48 (0.54)	21.06 (0.52)	69.55 (1.42)	
Gender,% (SE)				
Male	49.23 (0.31)	48.97 (0.32)	54.18 (1.38)	0.0003
Female	50.77 (0.31)	51.03 (0.32)	45.82 (1.38)	
Race,% (SE)				
Mexican American	8.50 (0.76)	8.61 (0.77)	6.46 (0.86)	<0.0001
Other Hispanic	5.21 (0.52)	5.33 (0.54)	2.78 (0.60)	
Non-Hispanic White	68.20 (1.51)	67.82 (1.52)	75.45 (1.05)	
Non-Hispanic Black	11.30 (0.80)	11.31 (0.80)	11.04 (1.05)	
Other Races	6.79 (0.40)	6.93 (0.41)	4.27 (0.75)	
Education level,% (SE)				
Less than high school	18.02 (0.66)	17.26 (0.65)	32.53 (1.59)	<0.0001
High school or GED	23.39 (0.53)	23.19 (0.54)	27.25 (1.27)	
Above high school	58.52 (0.96)	59.49 (0.96)	40.11 (1.86)	
Others	0.06 (0.02)	0.06 (0.02)	0.11 (0.07)	
Smoking status,% (SE)				
Never	52.87 (0.63)	53.61 (0.64)	38.76 (1.46)	<0.0001
Ever	23.47 (0.48)	22.71 (0.50)	38.04 (1.57)	
Current	20.92 (0.51)	20.82 (0.52)	22.72 (1.22)	
Unknown	2.74 (0.14)	2.86 (0.15)	0.48 (0.21)	
Diabetes,% (SE)	8.32 (0.23)	7.64 (0.22)	21.36 (1.20)	<0.0001
Hypertension,% (SE)	30.21 (0.52)	28.83 (0.53)	56.58 (1.51)	<0.0001
BMI (Kg/m2)	28.56 ± 0.08	28.57 ± 0.08	28.33 ± 0.16	0.1995
SBP (mmHg)	122.07 ± 0.22	121.44 ± 0.21	134.18 ± 0.69	<0.0001
DBP (mmHg)	70.55 ± 0.19	70.74 ± 0.19	66.80 ± 0.49	<0.0001
Urinary albumin (mg/L)	31.15 ± 1.52	25.93 ± 1.12	132.10 ± 20.50	<0.0001
Urinary creatinine (mg/dL)	121.87 ± 0.93	122.66 ± 0.95	106.68 ± 2.09	<0.0001
ALT (U/L)	25.65 ± 0.16	25.67 ± 0.15	25.36 ± 1.08	0.7753
AST (U/L)	25.79 ± 0.13	25.65 ± 0.11	28.54 ± 0.90	0.0014
Serum creatinine (mg/dL)	79.07 ± 0.26	78.07 ± 0.24	98.40 ± 2.01	<0.0001
Serum uric acid (μmol/L)	322.96 ± 0.88	321.65 ± 0.86	348.32 ± 3.27	<0.0001
Total cholesterol (mmol/L)	5.03 ± 0.01	5.04 ± 0.01	4.92 ± 0.04	0.0031
Triglycerides (mmol/L)	1.73 ± 0.02	1.73 ± 0.02	1.78 ± 0.04	0.2229
Fasting plasma glucose (uIU/mL)	5.83 ± 0.02	5.79 ± 0.02	6.61 ± 0.11	<0.0001
HDL-C (mmol/L)	1.37 ± 0.00	1.37 ± 0.01	1.37 ± 0.01	0.7395
LDL-C (mmol/L)	2.95 ± 0.01	2.96 ± 0.01	2.78 ± 0.04	<0.0001
WWI	10.89 ± 0.01	10.86 ± 0.01	11.46 ± 0.03	<0.0001
Follow-up time (month)	68.95 ± 1.07	69.97 ± 1.11	49.45 ± 0.90	<0.0001

GED, General Educational Development; BMI, Body Mass Index; SBP, Systolic Blood Pressure; DBP, Diastolic Blood Pressure; HDL-C, High Density Lipoprotein-Cholesterol; LDL-C, Low Density Lipoprotein-Cholesterol; ALT, Alanine Transaminase; AST, Aspartate Transaminase.

### *Follow-up and outcomes*

Over an average follow-up of 68.95 ± 1.07 months, 1870 participants died in total, 349 of cardiovascular causes. The all-cause mortality and cardiovascular mortality were 4.99% and 0.88% in the whole participants, respectively. Participants whose WWI < 11.3 showed much lower rates of all-cause mortality and cardiovascular mortality compared with those WWI ≥ 11.33 (All-cause mortality: 3.07% vs. 9.54%, *p* < 0.0001; Cardiovascular mortality: 0.49% vs. 1.81%, *p* < 0.0001). The prevalence of the causes of death were shown in Table 2.

**Table 2:** Outcomes among groups according to WWI levels (< 11.33 or ≥ 11.33).

	Total	WWI<11.33	WWI ≥11.33
No. (Unweighted)	26,882	17,731	9151
All-cause Mortality,% (SE)	4.99 (0.21)	3.07 (0.17)	9.54 (0.44)
Cardiovascular Mortality,% (SE)	0.88 (0.06)	0.49 (0.05)	1.81 (0.14)
Follow-up time, month (Mean ± SE)	68.95 ± 1.07	70.79 ± 1.13	64.60 ± 1.15
Cause of death			
Diseases of heart,% (SE)	15.94 (0.94)	14.56 (1.23)	16.99 (1.29)
Malignant neoplasms,% (SE)	23.26 (1.36)	23.97 (2.01)	22.72 (1.80)
Chronic lower respiratory diseases,% (SE)	1.93 (0.35)	1.65 (0.69)	2.14 (0.52)
Accidents (unintentional injuries),% (SE)	1.69 (0.37)	3.20 (0.66)	0.53 (0.36)
Cerebrovascular diseases,% (SE)	1.78 (0.49)	1.56 (0.69)	1.95 (0.55)
Alzheimer's disease,% (SE)	1.17 (0.21)	1.41 (0.49)	0.99 (0.38)
Diabetes,% (SE)	0.95 (0.31)	0.59 (0.35)	1.22 (0.47)
Influenza and pneumonia,% (SE)	0.23 (0.13)	0.37 (0.26)	0.13 (0.13)
Nephritis, nephrotic syndrome and nephrosis,% (SE)	0.64 (0.13)	0.55 (0.26)	0.71 (0.29)
Others,% (SE)	52.41 (1.69)	52.13 (2.37)	52.61 (2.03)

### *WWI could predict cardiovascular and all-cause mortality independently*

**Fig. 2:**
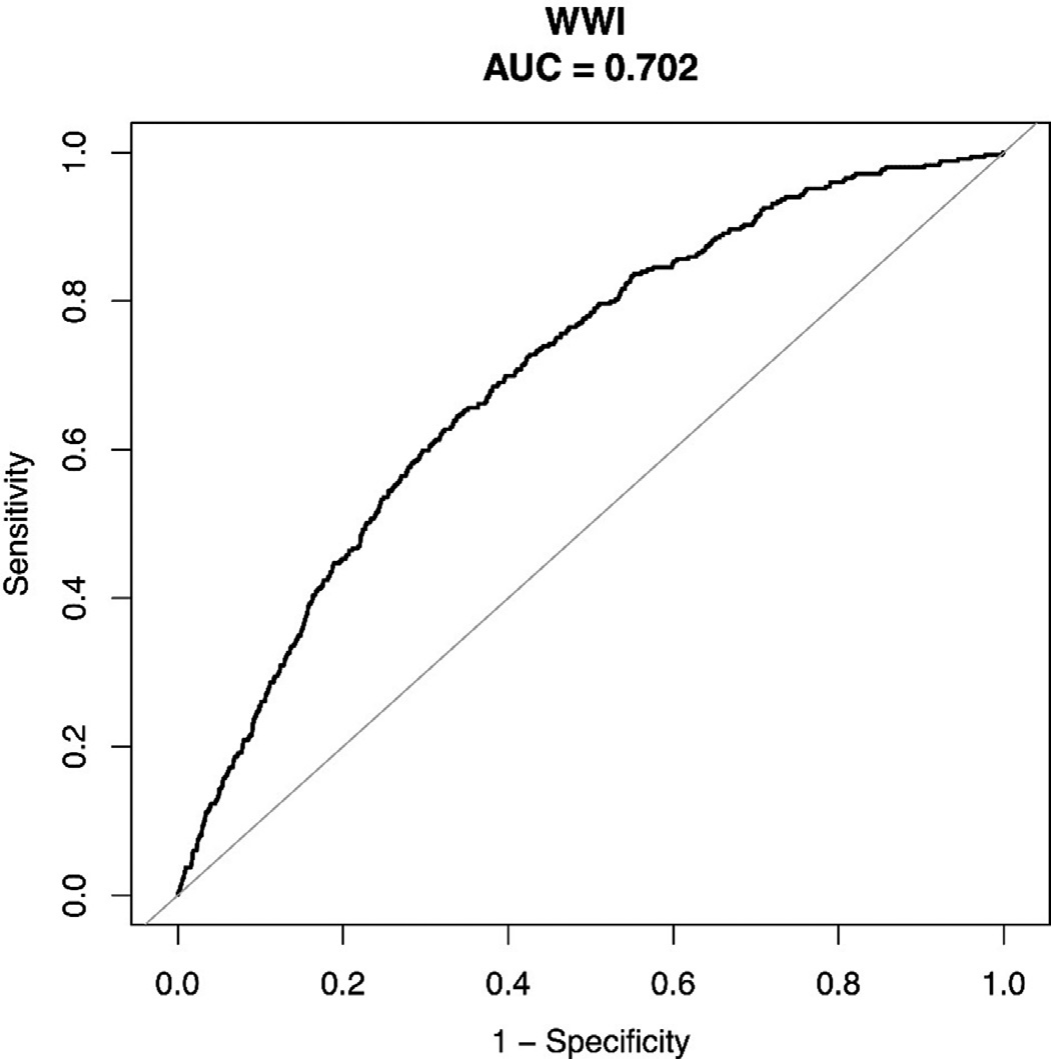
Receiver Operating Characteristic Curve (ROC) with Area Under the Curve (AUC) for WWI to discriminate the occurrence of cardiovascular death.

**Fig. 3:**
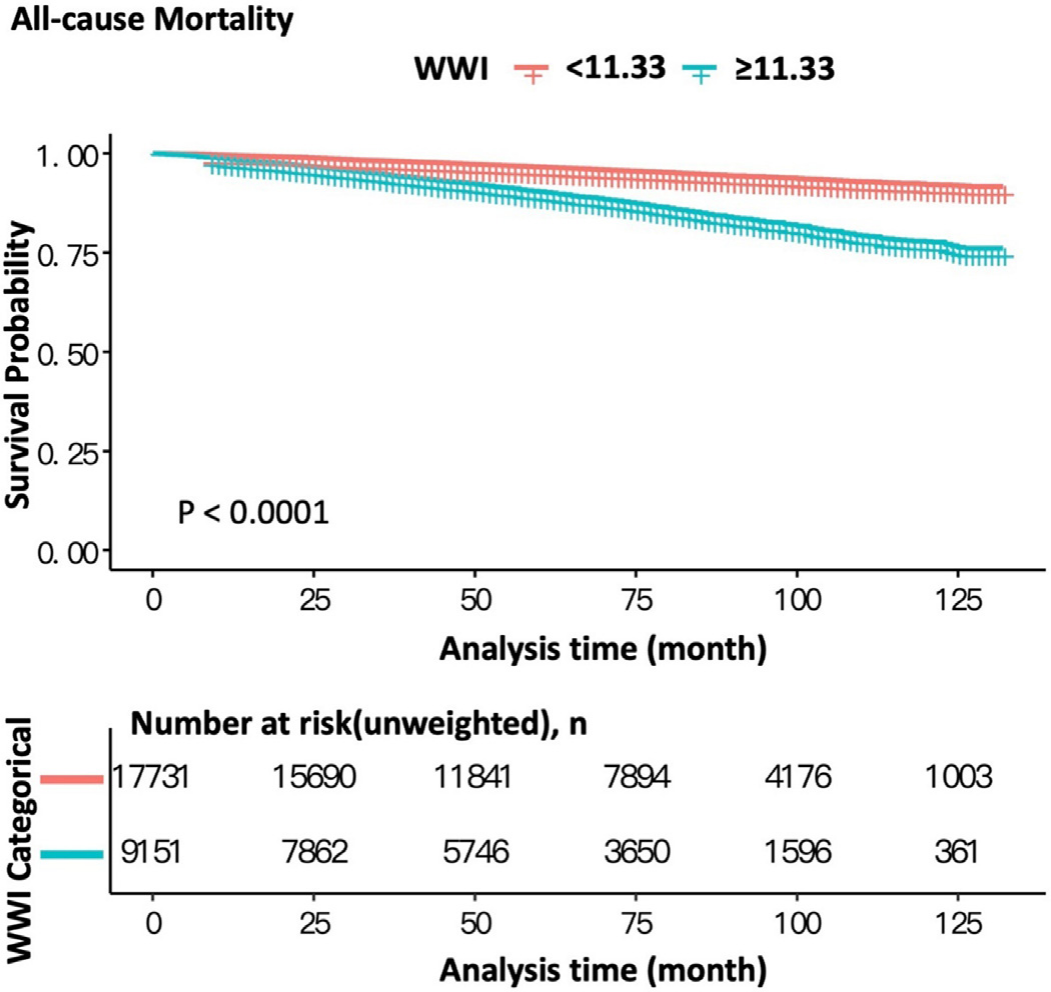
Kaplan-Meier survival curves of all-cause mortality according to WWI.

**Fig. 4:**
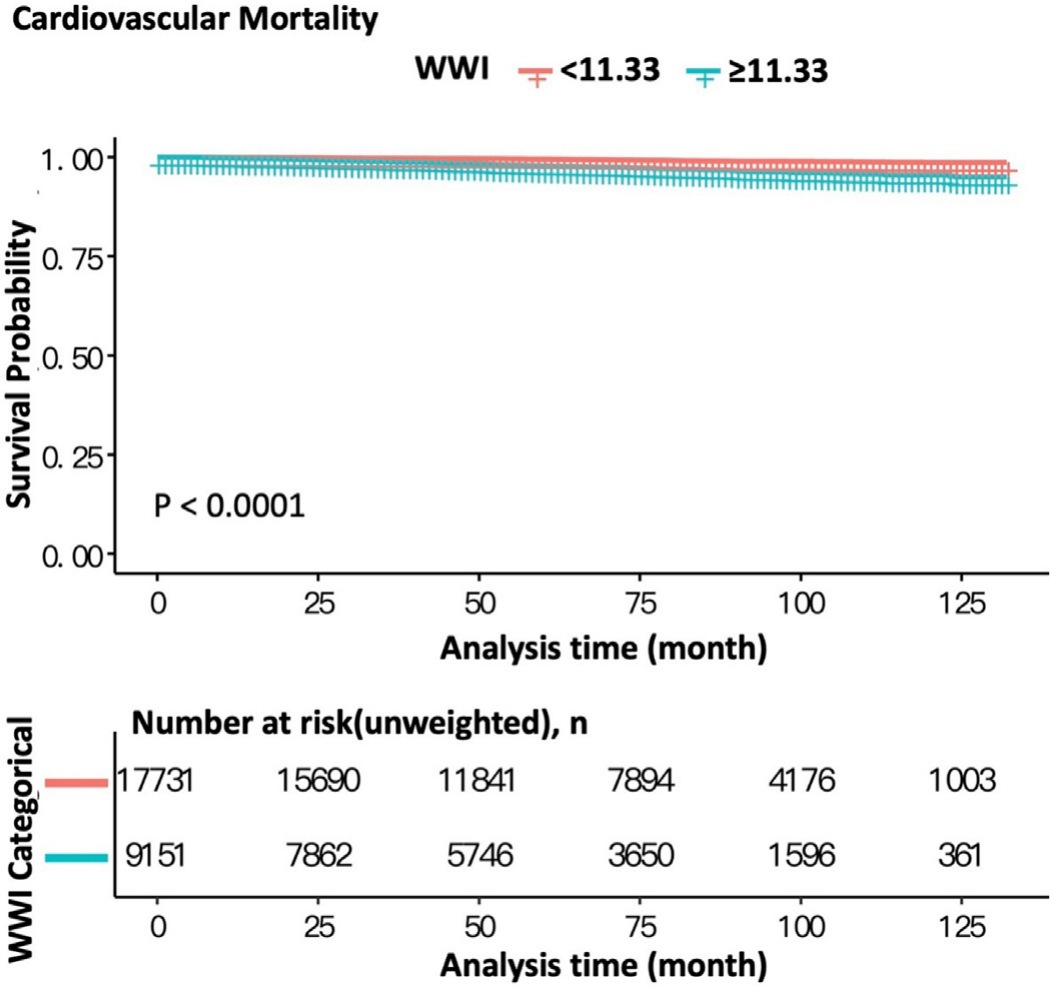
Kaplan-Meier survival curves of cardiovascular mortality according to WWI.

According to the ROC curve for the occurrence of cardiovascular death (Fig. 2), WWI presents a potential predictor for cardiovascular mortality, respectively (AUC = 0.702, specificity = 0.6623, sensitivity = 0.6447). The Kaplan-Meier analysis demonstrated significant differences in all-cause mortality and cardiovascular mortality between patients with WWI < 11.33 and ≥ 11.33 (both log-rank test *p* < 0.0001) with the adjustment of all variables (Figs. 3 and 4). These observations are also confirmed by the results of Cox proportional hazard regression analysis (Table 3). In the crude model, participants with WWI ≥ 11.33 showed a significant increased risk of CVD (HR = 3.73, 95% CI 2.99, 4.64) and all-cause death (HR = 3.03, 95% CI 2.76, 3.32) than the counterparts. These positive associations still remained statistically significant in the minimally adjusted model. After full adjustment, the authors observed that participant with WWI ≥11.33 had a 95% increased risk for cardiovascular death (HR = 1.95, 95% CI 1.30, 2.93) and 68% increased risk for all-cause death (HR = 1.68, 95% CI 1.41, 2.00) compared with those whose WWI < 11.3. The present results indicated that higher WWI was an independent risk factor and predictor for CVD and all-cause death in general US adults.

**Table 3:** Cox Proportional Hazard analysis for CVD and All-cause Mortality by according to WWI levels (< 11.33 or ≥ 11.33).

	HR (95% CI) WWI ≥ 11.33 versus <11.33
Cardiovascular Mortality	
Death, No./total No.	349/26,882
Crude model (Model 1)	3.73 (2.99, 4.64)
Minimally adjusted model (Model 2)	1.78 (1.41, 2.23)
Fully adjusted model (Model 3)	1.95 (1.30, 2.93)
All-cause Mortality	
Death, No./total No.	1870/26,882
Crude model (Model 1)	3.03 (2.76, 3.32)
Minimally adjusted model (Model 2)	1.56 (1.41, 1.72)
Fully adjusted model (Model 3)	1.68 (1.41, 2.00)

HR, Hazard Ratio; 95% CI, 95% Confidence Interval; Model 1, No covariates were adjusted; Model 2, Adjusted for gender, age and race; Model 3, Adjusted for gender, age, race, education level, urinary albumin, urinary creatinine, ALT, AST, total cholesterol, serum creatinine, triglycerides, serum uric acid, BMI, SBP, DBP, fasting plasma glucose, HDL-C, LDL-C, smoking, hypertension and diabetes status.

## Discussion

The objective of this study was to assess the correlation between WWI and cardiovascular mortality as well as all-cause mortality among non-institutionalized civilians in the United States. The present findings suggested that WWI may serve as a prognostic indicator for both CVD and all-cause death in the adult population. Participants with WWI ≥11.33 tended to have a higher risk of both cardiovascular mortality (HR = 1.95, 95% CI 1.30, 2.93) and all-cause mortality (HR = 1.68, 95% CI 1.41, 2.00) compared with those who have lower WWI. Overall, the results suggested that WWI could be an independent predictor for mortality among US adults.

Previous studies also reported a similar association in different population settings. In 2018, WWI was initially proposed by Park et al.^5^ Their research demonstrated that WWI exhibits a strong predictive capacity for both cardiometabolic morbidity and mortality among the Korean population. Furthermore, serving as a comprehensive predictor, WWI exhibits a significant positive correlation with all prognoses related to cardiometabolic disease morbidity and mortality, whereas BMI and WC do not demonstrate such a relationship. Ding and colleagues discovered a non-linear positive association between WWI with cardiovascular mortality and all-cause in southern China, which remained statistically significant even after controlling for BMI and WC.^11^ Their study revealed a significant association between elevated WWI levels exceeding 11.2 and an augmented risk of mortality. The present findings are consistent with the majority of the current literature, which posits that heightened WI is a detrimental factor for patient health and prognosis.

The relationships between other physical measures to assess obesity and prognosis have also been reported. At present, BMI stands as the predominant measure employed for categorizing obesity. Individuals with an elevated BMI are at an increased risk of all-cause mortality.^3^ Elevated BMI not only poses a heightened risk for all-cause mortality, but also portends an unfavorable prognosis for a range of chronic ailments, such as hypertension, heart disease, gallstones, colon cancer, and stroke as well. WC, which refers to the horizontal circumference through the center of the umbilicus, is a composite indicator of total adiposity and fat distribution. The findings of a Chinese cohort study indicate a significant correlation between the prevalence of dyslipidemia in a rural Chinese population and the dynamic changes in WC.^12^ Reducing WC may lower the risk of dyslipidemia, while increasing WC may increase the risk. Visceral Adiposity Index (VAI), which is determined by the measurement of WC, BMI, Triglycerides (TG), and High-Density Lipoprotein cholesterol (HDL-c), serves as an indirect indicator of visceral adipose function.^13^^,^^14^ According to a recent retrospective analysis, the VAI demonstrated significant value as an indicator for both predicting and evaluating the severity of Hyperlipidemic Acute Pancreatitis (HLAP).^15^ They found that patients diagnosed with severe and moderate Acute Pancreatitis (AP) exhibited a significantly higher value of VAI compared to those with mild AP. Furthermore, the results revealed a strong correlation between elevated VAI levels and unfavorable prognosis in patients diagnosed with HLAP. The Waist-to-Hip Ratio (WHR) has been linked to visceral adiposity and may serve as an indicator of the extent of central adiposity. Studies have shown that WHR is an independent risk factor for the long-term prognosis of Chinese individuals who have undergone revascularization for coronary heart disease. In the long term, patients exhibiting a higher WHR demonstrated an increased likelihood of developing Major Adverse Cardiac Events (MACEs).^16^ The concept of the Waist-to-Height Ratio (WHtR) was developed to improve the WHR. The evaluation of abdominal obesity is accomplished through the utilization of the WHtR, which takes into account the individual's height in relation to their WC, thereby rectifying the limitations of the WHR. As such, WHtR surpasses WC and BMI as more effective indicators.^17^^,^^18^ The study conducted by Chen and his colleagues revealed a positive correlation between WHtR and higher mortality risk in individuals diagnosed with Heart Failure with preserved Ejection Fraction (HFpEF). The results indicated that an elevated WHtR was an independent risk factor for all-cause mortality in Chinese patients with HFpEF.^19^ These obesity assessment metrics are similar to WWI in this study. However, compared with other indicators, using WWI as an assessment indicator is easier to perform, less costly and non-invasive to the human body.

The underlying mechanisms that account for the association between elevated WWI and poor prognosis remain inadequately comprehended. Numerous hypotheses have been proposed to account for the unfavorable prognostic implications of obesity, including concentrations of circulating hormones such as estrogen and androgens, reduced levels of sex hormone-binding globulin which elevate free estradiol and free testosterone, elevated levels of insulin and insulin-like growth factor, diminished levels of insulin-like growth factor binding protein, elevated levels of cortisol and leptin, augmented levels of cytokines, disrupted lipid metabolism, and heightened levels of inflammation culminating in compromised immune function.^20^^,^^21^ The main mechanisms of poor prognosis due to obesity represented by increased WWI may be the disorders of lipid metabolism.^22^ Disorders of lipid metabolism are abnormalities of lipids and lipid metabolites present mainly in plasma and other tissues. Increased WWI reflects adipose tissue dysfunction, indicating abnormal lipid levels and disorders of lipid metabolism. Numerous studies have shown that disorders of lipid metabolism were associated with cancer prognosis. Carcinogenesis and cancer metastasis have a high demand for energy, which induces alterations in lipid metabolism, thus allowing cancer cells to survive. Secondly, alterations in microvascular structure and function may also contribute to the poor prognosis.^23^ Obesity exerts an impact on the microvascular system of multiple tissues, resulting in structural and functional alterations that impair the proper function of the corresponding tissues and organs. This, in turn, leads to a dysregulated release of inflammatory cytokines and adipocytokines, including leptin, lipocalin, and TGF-beta, which contributes to the development of oxidative stress, inflammatory response, endothelial dysfunction, and microvascular remodeling.

The present research exhibits multiple strengths. First, the sample size of the study subjects was large and representative, and the follow-up period was long. Second, the exposure data of the study subjects were collected before the occurrence of the outcome and were obtained by personal observation of the investigators, and the recall bias was relatively small. Finally, this analysis was adjusted for some confounding factors to make the results more reliable. However, limitations in the analysis could not be ignored. Despite adjusting for certain potential covariates, the influence of other possible confounding factors, such as the use of medications including diuretics and steroids, could not be entirely eliminated. Furthermore, it should be noted that the individuals included in the present analysis were solely from a single country, and as such, their ethnic background may not be representative of a diverse range of countries globally. Consequently, the present research outcomes cannot be extrapolated to the wider population or to other ethnic groups, thereby restricting the generalizability of the findings.

## Conclusion

The present study demonstrated that elevated WWI levels were associated with a higher risk of cardiovascular mortality and all-cause mortality. The present findings suggested that WWI could potentially function as a simple and efficacious anthropometric index for forecasting the incidence of cardiovascular and all-cause death. However, additional studies are still needed to authenticate the present findings.

## Data availability statement

Publicly available datasets were analyzed in this study. These data can be found at: www.cdc.gov/nchs/nhanes/.

### Ethics statement

The studies involving human participants were reviewed and approved by the National Center for Health Statistics. The patients/participants provided written informed consent to participate in this study.

## CRediT authorship contribution statement

**Ying Han:** Investigation, Writing – original draft. **Jieli Shi:** Software, Conceptualization, Writing – review & editing. **Pengfei Gao:** Data curation. **Lin Zhang:** Methodology. **Xuejiao Niu:** Validation. **Na Fu:** Supervision.

## Declaration of Competing Interest

The authors declare no conflicts of interest.
